# Down-Regulation of miR-92 in Human Plasma Is a Novel Marker for Acute Leukemia Patients

**DOI:** 10.1371/journal.pone.0005532

**Published:** 2009-05-14

**Authors:** Masami Tanaka, Kosuke Oikawa, Masakatsu Takanashi, Motoshige Kudo, Junko Ohyashiki, Kazuma Ohyashiki, Masahiko Kuroda

**Affiliations:** 1 Department of Pathology, Tokyo Medical University, Tokyo, Japan; 2 Department of Cell Therapy, Tokyo Medical University, Tokyo, Japan; 3 Intractable Disease Therapeutic Research Center, Tokyo Medical University, Tokyo, Japan; 4 First Department of Internal Medicine, Tokyo Medical University, Tokyo, Japan; Institute of Cancer Research, United Kingdom

## Abstract

**Background:**

MicroRNAs are a family of 19- to 25-nucleotides noncoding small RNAs that primarily function as gene regulators. Aberrant microRNA expression has been described for several human malignancies, and this new class of small regulatory RNAs has both oncogenic and tumor suppressor functions. Despite this knowledge, there is little information regarding microRNAs in plasma especially because microRNAs in plasma, if exist, were thought to be digested by RNase. Recent studies, however, have revealed that microRNAs exist and escape digestion in plasma.

**Methodology/Principal Findings:**

We performed microRNA microaray to obtain insight into microRNA deregulation in the plasma of a leukemia patient. We have revealed that microRNA-638 (miR-638) is stably present in human plasmas, and microRNA-92a (miR-92a) dramatically decreased in the plasmas of acute leukemia patients. Especially, the ratio of miR-92a/miR-638 in plasma was very useful for distinguishing leukemia patients from healthy body.

**Conclusions/Significance:**

The ratio of miR-92a/miR-638 in plasma has strong potential for clinical application as a novel biomarker for detection of leukemia.

## Introduction

microRNAs are small endogenous non-coding RNAs that posttranscriptionally repress the expression of protein-coding genes by base-pairing with the 3′ untranslated regions (UTRs) of the target messenger RNAs (mRNAs) [1]. These single-strand RNAs are considered to play crucial roles in many normal cellular processes, such as proliferation, development, differentiation and apoptosis by inhibiting target gene(s) expression through imperfect pairing with target mRNAs, inducing direct mRNA degradation or translational inhibition [2], [3], [4], [5]. In addition, recent studies have shown that the deregulation of microRNA expression contributes the multistep processes of carcinogenesis in human cancer either by oncogenetic or tumor suppressor function [6], [7]. Therefore, microRNA expression patterns (or signatures) are now known to characterize the developmental origins of tumors more effectively than mRNA expression signatures and may provide a useful tool for the diagnosis and prognosis of human cancer.

The search for non-invasive tools for the diagnosis and management of cancer has long been a goal of cancer research, and it could greatly reduce the worldwide health burden of cancer [8]. Although conventional strategies for blood-based biomarker discovery (e.g., using proteomic technologies) have shown promise, novel approaches that can complement and improve on current strategies for cancer detection are urgently needed. Recently, it has been reported that microRNAs are circulating in serum [9], [10] and tumor-derived microRNAs such as miR-155, miR-21, miR-15b, miR-16 and miR-24 are detected in the plasmas and serums of tumor patients [11], [12]. These might be a new class of effective biomarkers, and we expect that the microRNAs abundance profile in plasma might reflect physiological and/or pathological conditions.

In this study, we focused whether circulating microRNAs can be detected in plasma and whether expression levels of specific microRNAs differ between leukemia patients [several type of acute myeloid leukemia (AML) and acute lymphoblastic leukemia (ALL)] and healthy individuals. Our results have revealed that the decrease of miR-92a in the plasma is a novel class of blood-based leukemia biomarkers, and furthermore raised provocative questions regarding the mechanism of stability and potential biological function of circulating microRNAs.

## Results

### Expression profiling of microRNAs in normal human plasma

**Figure 1:**
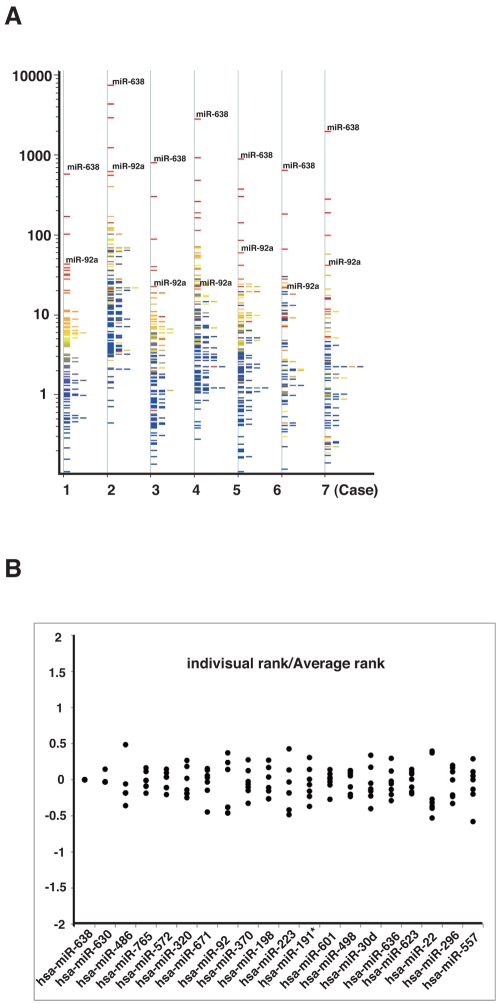
Expression profiling of microRNAs in normal plasmas. A. MicroRNA expression profiles in seven normal samples by microRNA microarray analysis. Y axis represents relative intensity of hybridization signals. B. Comparison of the signal intensities of various microRNAs among normal plasmas. The signal intensity of each microRNA by microarray analysis is evaluated as a rank order among the detected microRNAs. Y axis represents log_10_ (Rank of signal intensity in each sample/average rank of that in all samples).

Mitchell et al. have reported that ninety-one mature microRNAs are present in human plasma, and the endogenous plasma microRNAs might exist in a form that is resistant to plasma RNase activity [12]. Therefore, we also analyzed the expression profile of microRNAs in human plasma using microarrays. We found that 148 microRNAs presented in seven normal plasma samples (Figure 1A). Unfortunately, it was difficult to compare these signal intensities because there was great variability of the values among the samples. However, the rank order of signal intensity of each microRNA among all of the detected microRNAs in a sample was similar to that in the other samples (Figure 1B). Especially, the signal of miR-638 was the top rank regardless of the sexes and ages. These data suggested that miR-638 was physiologically necessary in blood, and might be a useful internal control for quantification of microRNAs in plasma. Our results of the microRNA expression profiles were somewhat different from previously reported data [10], [12]. The difference may be possible to owe to the different technical platform.

### Microarray analysis of the microRNAs in the plasma of leukemia patients

**Figure 2:**
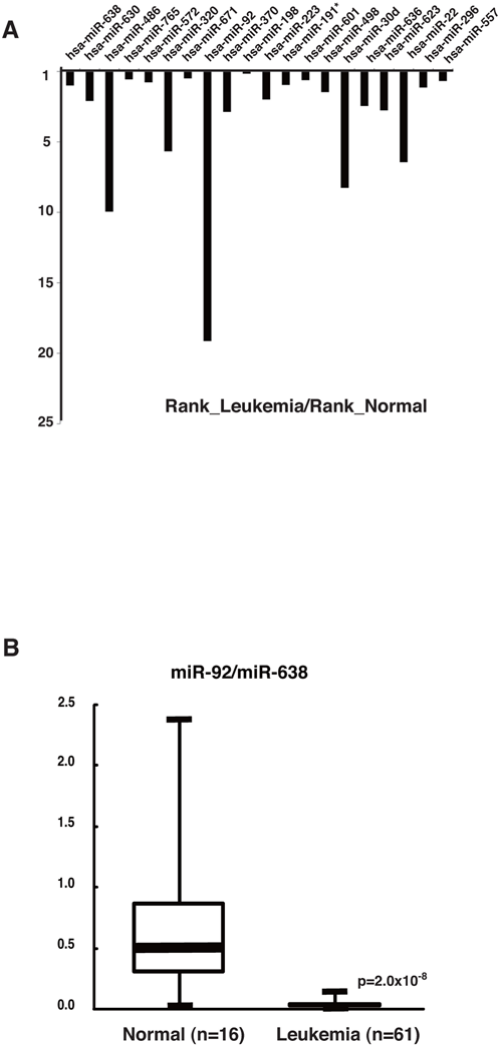
Comparison of microRNA expressions in the plasmas of normal and acute leukemia. A. Comparison of signal intensity ranks of various microRNAs in normal (n = 7) and leukemia (n = 2) plasmas by microarray analysis. Y axis represents mean ratio of the signal intensity rank of leukemia to the rank of normal of each microRNA. B. Comparison of the ratio of miR92a signal intensity to miR-638 signal intensity by *Taq*Man qRT-PCR among the plasmas of normal and leukemia. Mann-Whitney's U test was used to determine statistical significance.

Above results led us to determine whether tumor-related microRNAs enter the circulation at levels sufficient to be measurable as biomarkers for leukemia detection. To identify the differentially expressed microRNAs in the plasmas of acute leukemia patients, we first screened leukemia samples using microarrays. Experiments were performed with total RNA isolated from the plasmas of two acute myeloid leukemia (AML) leukemia samples. We found that the rank order of signal intensities of miR-92a was dramatically down in the leukemia samples (Figure 2A).

### The ratio of miR-92a and miR-638 serves as leukemia biomarkers

We next analyzed the levels of miR-92a in the plasma samples from normal (n = 16) and leukemia (AML, n = 54; ALL, n = 7) by *Taq*Man qRT-PCR. In order to improve the precision of the data, we tried to use the miR-638 as standardization. Interestingly, the ratio of miR-92a to miR-638 in all of the plasma samples from the leukemia patients were decreased compared with that from the normal donors (Figure 2B).

### High expression of miR-92a in tumor cells of acute leukemia

**Figure 3:**
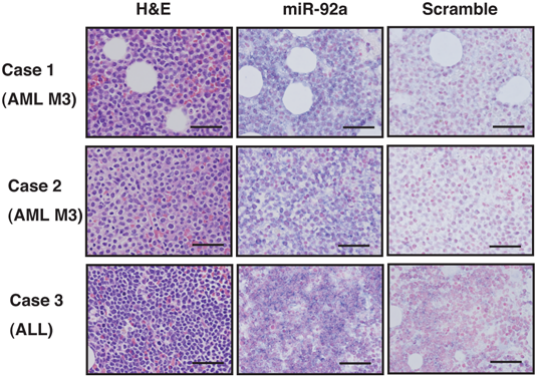
MicroRNA expression in leukemic cells. *In situ* hybridization was performed using LNA probes for miR-92a and negative control. Blue signals represent positive for the microRNAs. Bars indicate 50 µm.

Next, we examined the expression level of miR-92a in leukemic cells. We performed *in situ* hybridization using locked nucleic acid (LNA)-modified probes digoxigenin (DIG) labelled. We examined 4 cases of AML and 2 cases of ALL, and found that miR-92a was strongly expressed in leukemic cells from both AML and ALL (Figure 3, Case 1–3). In contrast, we did not detect miR-92a expression in normal blasts.

## Discussion

In this report, we describe that the ratio of miR-92a/miR-638 in plasma was the very sensitive marker for AML and ALL. There was no relationship between the subtypes of AML and ALL (data not shown). Recently, microRNA expression profiling studies revealed that t(8;21), inv(16), t(15;17) and MLL/11q23 have unique microRNA expression signatures capable of distinguishing them from other subtypes of AML [13], [14], [15]. In addition, it has been reported that up-regulation of miR-155 in AML patients with an internal tandem duplication (ITD) of the FLT3 gene [13], [16], [17]. These observations suggest that microRNA expression is associated with cytogenetics and molecular alterations in AML. On the other hand, we observed decrease of miR-92a in all plasma samples of AML and ALL. One case in the samples showing the miR-92a decrease has t(4;11)(q21;q23) translocation. These data indicate that microRNA expression signature in plasma will serve as a valuable diagnostic and prognostic marker that adds information beyond the cytogenetics. The decrease of miR-92a in the plasma may be a very useful clinical marker of acute leukemia.

What are the physiological roles of miR-638 and miR-92a in the blood vessels and why miR-92a is decreased in plasma of the acute leukemia patients are intriguing questions but remain unclear. Interestingly, miR-92a is transcribed from miR-17-92 locus that encode the polycistronic precursor containing seven microRNAs: miR-17-5p, miR-17-3p, miR-18, miR-19a, miR-20, miR-19b and miR-92a, and the human microRNA cluster miR-17-92 is amplified and/or overexpressed in several cancers such as acute myeloid leukemia [15], [18], malignant lymphoma [19], lung cancer [20], thyroid cancer [21] and hepatocellular carcinoma [22]. Furthermore, inhibition of miR-17-5p and miR-20a with antisense oligonucleotides (ONs) can induce apoptosis selectively in lung cancer cells overexpressing miR-17-92 [23]. All together, these results suggest that miR-92 has oncogenic potential. Interestingly, we revealed that discrepant expression of miR-92a between leukemic cells and the plasmas of leukemic patients. At the time we speculate that miR-92a is an essential molecule for proliferation of cells, and cancer cells both actively transcribe miR-92a and take in miR-92a from the blood. Exosomes are small (50–90 nm) membrane vesicles of endocytic origin that are released into the extracellular environment on fusion of multivesicular bodies (MVB) with the plasma membrane [24]. Many cells including reticulocytes [25], dendritic cells [26], B cells [27], T cells [28], mast cells [29], epithelial cells [30] and tumor cells [31] have the capacity to release exosomes, and exosomes contain both mRNAs and microRNAs, which can be delivered to another cell and function in a new location [32]. In addition, endogenous plasma microRNAs exist in a form that is resistant to RNase activity in plasma [12]. These studies suggest that microRNAs are packaged inside exosomes that are secreted from cells. Thus, it might be possible that cancer cells specifically take in the exosome that contain miR-92a and, as a result, miR-92a decreases from the blood. As an alternative explanation, cancer cells may specifically digest miR-92a in the plasma directly or indirectly. Nevertheless, it is still unclear that selective decrease of miR-92a in acute leukemia, and we need to study the mechanism of microRNA circulation. Future studies may reveal how these microRNAs influence primate physiology. In addition, many microRNAs expressed in tumor tissues may influence the biological behavior and clinical phenotype of the tumor. Although the functional roles of microRNAs in tumor biology are unrevealed, we envision that blood-based microRNA biomarkers will predict clinical behavior and/or therapeutic response.

In summary, we have shown that the ratio of miR-92a/miR-638 in blood is firmly associated with diagnosis in acute leukemia patients. This data suggest that the amount of microRNAs in plasma have potential as non-invasive biomarkers of cancer, including hematopoietic neoplasia.

## Materials and Methods

### Plasma collection and RNA isolation

Whole blood samples were collected from healthy donors and the patients with AML and ALL at Tokyo Medical University Hospital (Tokyo, Japan). This study was approved in June 2008 by institutional review board (IRB) of Tokyo Medical University (No. 930), and all subjects provided written informed consent under institutional review board. Details of clinical data are provided in Table 1. Whole blood was separated into plasma and cellular fractions by centrifugation at 1600×g for 15 min. Total RNA in the plasma was isolated using Isogen-LS (NIPPON GENE) according to the manufacture's instructions. For RNA isolation from plasma, 250 ml of plasma was homogenized in 750 ml of Isogen-LS. Then 200 µl of chloroform was added to the sample and the mixed solution was centrifugated. After an additional chloroform extraction and precipitation with isopropanol, the RNA sample was suspended in 20 µl of nuclease free water. In general, we obtained 400 ng of RNA from 1 ml of plasma.

**Table 1:** Summary of clinical detalis of acute leukemia patients used for analysis.

	n(%)
Sex	
Men	39(51%)
Women	38(49%)
Total	77(100%)
Control	16(19%)
French-American-British classification	
AML M0	2(3.2%)
AML M1	11(18.0%)
AML M2	19(31.1%)
AML M3	10(16.3%)
AML M4	3(4.9%)
AML M4Eo	1(1.6%)
AML M5	1(1.6%)
AML M5b	1(1.6%)
AML M6	1(1.6%)
AML M7	2(3.2%)
AML with multilineage dysplasia	3(4.9%)
ALL L2	2(3.2%)
ALL Ph +	1(1.6%)
ALL preB	4(6.5%)
Acute leukemia Total	61(100%)

### MicroRNA microarray analysis

To monitor the changes in microRNA levels associated with AML, 100 ng of total RNA was labeled and hybridized using Human microRNA Microarray Kit (Agilent Technologies) according to manufacture's protocol (Protocol for use with Agilent microRNA microarrays Version 1.5). Hybridization signals were detected with DNA microarray scanner (Agilent Technologies) and the scanned images were analyzed using Agilent feature extraction software. The data discussed in this manuscript have been deposited in NCBI's Gene Expression Omnibus and are accessible through GEO Series accession number GSE14772: http://www.ncbi.nlm.nih.gov/geo/query/acc.cgi?token=tdabvuqcakymoxi&acc=GSE14772.

### Quantitative RT-PCR of mature microRNAs

MicroRNAs were quantified using TaqMan® MicroRNA Assays (Applied Biosystems) with modifications. Briefly, 20 ng of total RNA was reverse transcribed (RT) by TaqMan® MicroRNA RT Kit. 15 µl of RT reactions contained 10× RT buffer, 0.15 µl of 100 mM dNTPs with dTTP, 0.188 µl of RNase-inhibitor (20 units/µl), 1 µl of MultiScribe™ Reverse Transcriptase (50 units/µl), 1 µl of each of microRNA specific stem-loop primers (has-miR-92, 4374013; has-miR-638, 4380986; Applied Biosystems) and 10.16 µl of input RNA. The mixture was incubated at 16°C for 30 min, 42°C for 30 min and 85°C for 5 min. Subsequently, quantitative real time-PCR was performed using Mx3005P™ QPCR System (STRATAGENE). For a 20 µl of PCR reaction, 20× TaqMan® MicroRNA Assays in which PCR primers and probes (5′-FAM and 3′-TAMRA) were contained, 1 µl of RT product diluted 1∶2 with nuclease-free water and 10 µl of 2× TaqMan® Universal Master Mix, No AmpErase® UNG (Applied Biosystems) were mixed. The reaction was first incubated at 95°C for 2 min, followed by 50 cycles of 95°C for 15 sec and 60°C for 1 min. Data were analyzed with MxPro–Mx3005P version 3.00 (STRATAGENE), with the automatic Ct setting for adapting baseline and threshold for Ct determination. Mann-Whitney's U test was used to determine statistical significance between control and test group. *P* values less than 0.05 were considered significant.

### In situ hybridaization of miR-92a

Locked nucleic acid (LNA)-modified probes for miR-92a and negative control (miRCURY-LNA detection probe, Exiqon). The probe sequences were as follows; *miR-92a*, 5′-ACAGGCCGGGACAAGTGCAATA-3′; a scrambled oligonucleotides used for negative control, 5′-GTGTAACACGTCTATACGCCCA-3′.

*In situ* hybridization was performed using the RiboMap *in situ* hybridization kit (Ventana Medical Systems) on the Ventana Discovery automated *in situ* hybridization instrument (Ventana Medical Systems). The i*n situ* hybridization steps after the deparaffinization step were performed based on the standard protocol provided in the manufacturer's RiboMap application note (http://www.ventanamed.com). The initial fixation step was performed by incubating the sections in formalin-based RiboPrep reagent (Ventana Medical Systems) for 30 min at 37°C. After acid treatment using hydrochloride-based RiboClear reagent (Ventana Medical Systems) for 10 min at 37°C, the slides were treated with the ready-to-use protease 2 reagent. The sections were hybridized with the anti-sense LNA (Locked Nucleic Acid) riboprobe (2 ng/slide) using RiboHybe hybridization buffer (Ventana Medical Systems) for 6 h at 37°C after an initial denaturing pre-hybridization step for 6 min at 70°C. After a low-stringency wash with 2× RiboWash (Ventana Medical Systems) for 6 min at 42°C, a second washing step was performed using 1× RiboFix reagent for 20 min at 37°C followed by incubation of biotin-labeled anti-digoxigenin antibody (Sigma) for 30 min at 37°C. After incubating the streptavidin-alkaline phosphatase conjugate for 16 min at 37°C, signal was detected using the BlueMap NBT/BCIP substrate kit for 4 h at 37°C. Finally, the sections were counterstained with Kernechtrot as a marker stain and covered with a glass coverslip.
